# A Review of Road Traffic-Derived Non-Exhaust Particles: Emissions, Physicochemical Characteristics, Health Risks, and Mitigation Measures

**DOI:** 10.1021/acs.est.2c01072

**Published:** 2022-05-25

**Authors:** Julia C. Fussell, Meredith Franklin, David C. Green, Mats Gustafsson, Roy M. Harrison, William Hicks, Frank J. Kelly, Franceska Kishta, Mark R. Miller, Ian S. Mudway, Farzan Oroumiyeh, Liza Selley, Meng Wang, Yifang Zhu

**Affiliations:** † National Institute for Health Research Health Protection Research Unit in Environmental Exposures and Health, School of Public Health, Imperial College London, London, W12 0BZ, U.K.; ‡ Department of Statistical Sciences, University of Toronto, Toronto, Ontario M5G 1Z5, Canada; § Swedish National Road and Transport Research Institute (VTI), SE-581 95, Linköping, Sweden; ∥ School of Geography, Earth and Environmental Sciences, University of Birmingham, Birmingham, B15 2TT, U.K.; ⊥ Centre for Cardiovascular Science, Queen’s Medical Research Institute, University of Edinburgh, Edinburgh, EH16 4TJ, U.K.; # Department of Environmental Health Sciences, Jonathan and Karin Fielding School of Public Health, University of California, Los Angeles, Los Angeles, California 90095, United States; ∇ MRC Toxicology Unit, University of Cambridge, Gleeson Building, Tennis Court Road, Cambridge,CB2 1QR, U.K.; ¶ University at Buffalo, School of Public Health and Health Professions, Buffalo, New York 14214, United States; ⬡ Department of Environmental Sciences / Centre of Excellence in Environmental Studies, King Abdulaziz University, Jeddah, 21589, Saudi Arabia

**Keywords:** exposure assessment, health effects, mitigation, nonexhaust emissions, road traffic, toxicity

## Abstract

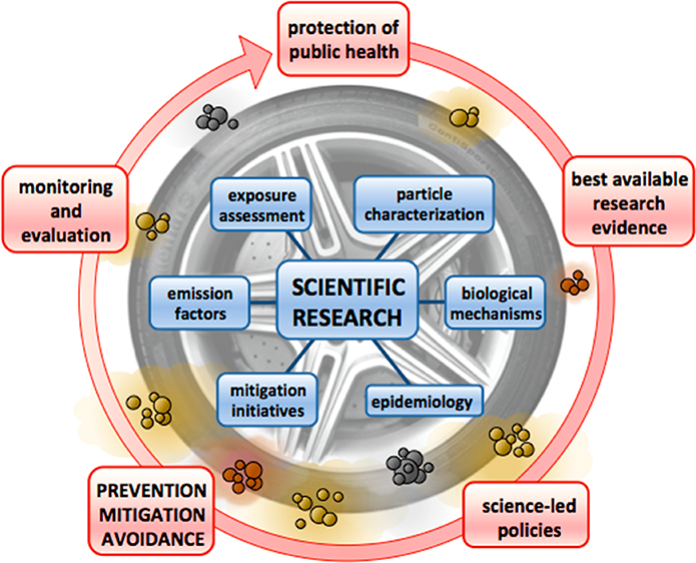

Implementation of regulatory standards has reduced exhaust emissions of particulate matter from road traffic substantially in the developed world. However, nonexhaust particle emissions arising from the wear of brakes, tires, and the road surface, together with the resuspension of road dust, are unregulated and exceed exhaust emissions in many jurisdictions. While knowledge of the sources of nonexhaust particles is fairly good, source-specific measurements of airborne concentrations are few, and studies of the toxicology and epidemiology do not give a clear picture of the health risk posed. This paper reviews the current state of knowledge, with a strong focus on health-related research, highlighting areas where further research is an essential prerequisite for developing focused policy responses to nonexhaust particles.

## 1 Introduction

A compelling body of evidence exists associating air pollution with increased morbidity and mortality from cardiorespiratory disease and lung cancer, with further accumulating evidence for diabetes, neurological damage, and adverse birth outcomes.^1^ The significance of the problem is reflected in the updated World Health Organization Global Air Quality Guidelines, which recently recommended substantially lower air quality limits for PM_2.5_, PM_10_ (particulate matter less than 2.5 and 10 μm in diameter respectively), and nitrogen dioxide.^2^ Increasing urbanization brings an ever-growing exposure to air pollutants from road traffic that is linked to acute and chronic health effects.^3^ Mechanistic studies in humans, animals and cells support epidemiological findings,^4^ demonstrating biological plausibility for multiorgan effects through an array of biological mechanisms.^5−8^

Traffic-derived air pollution comprises a mixture of gaseous pollutants and PM from fuel combustion and lubricant volatilization in exhaust (tailpipe) emissions. Road transport is also a source of nonexhaust emissions (NEE). These comprise of particles from mechanical abrasion of brakes and tires, erosion of road surfaces and resuspension of a mixture of dust that accumulates on road surfaces, and volatile organic compounds from evaporative loss of fuels and release of solvents.^9,10^ Most epidemiological and experimental research into traffic-related pollution focuses on particulate and gaseous pollutants emitted from the exhaust, particularly from diesel-fueled vehicles. In contrast, particulates from NEE have been woefully understudied. NEE, especially those from brake and tire wear, are an important source of metals in urban atmospheres.^11^ The UK emissions inventory estimates mass contributions of 47% and 21% to national total airborne emissions of Cu and Zn, respectively.^12^ These metals, as well as those such as Fe from brake wear, catalyze the formation of reactive oxygen species (ROS) in the respiratory tract lining fluids, challenging antioxidants and metal-binding proteins that protect the epithelial surface of the lung.^13^ While invariably being associated with coarse-mode PM, a considerable fraction of abrasion-derived particles exist within the fine and ultrafine fractions,^14,15^ engendering NEE with a high capacity for harm owing to a larger reactive surface area and the ability to penetrate deeper into the lung and possibly into the blood to impact other organs in the body.

In developed countries, tightening emission regulations for gasoline and diesel vehicles has mandated technological upgrades of combustion control and exhaust emission treatment systems. This has been effective in progressively driving down gaseous pollutants and PM from the exhaust of new vehicles. As a consequence, atmospheric emission inventories indicate that the proportion of NEE has increased,^10^ widely exceeding exhaust emissions.^12^ In the UK, 2016 emissions data from the National Atmospheric Emissions Inventory (NAEI) showed that nonexhaust particles are the main source of primary PM (by mass) from road transport, for both the PM_2.5_ (60%) and PM_10_ (73%) size fractions. A steady growth in this nonexhaust contribution is forecast, owing to phasing-out of older vehicles, increased electrification of road transport and the absence of legislation to limit/reduce nonexhaust particles. It is not surprising, therefore, that calls have been made for NEE from traffic to receive immediate recognition as an important source of ambient PM.^12^

Several reviews have been published on aspects of NEE;^9,10,12,16,17^ however, to our knowledge, previous articles have not incorporated health-related data, nor examined mechanisms driving NEE toxicity. Here we critically assimilate evidence on (a) the characterization of nonexhaust particles (Section 2), (b) their contributions to concentrations of, exposures to and health effects of ambient particulate matter (Sections 2–4), plus (c) the toxicological properties of this compositionally distinct source of particles (Section 5). We also address mitigation initiatives and identify critical research directions.

## 2 Emission Characteristics and Quantification

NEE can be characterized in the laboratory at the subsystem level by testing individual material composites and different vehicle speeds/drive cycles on dynamometers.^18−21^ Alternatively, laboratory and on-road testing at the system level utilize real cars and chassis to assess specific variables under controlled test conditions,^22−25^ providing controlled and well-defined emission estimates. However, they are unable to characterize the entire vehicle fleet and variations in driving styles, while climate and atmospheric processes may be relatively unrepresentative, causing discrepancies with real-world emissions.

Individual elemental tracers can be measured in the atmosphere and used to estimate NEE based on the material composition and/or scaling factors that account for real world emissions from the vehicle fleet under a range of meteorological conditions.^26−28^ However, this approach is potentially limited by wide variations in brake and tire material composites and is less able than laboratory studies to address specific vehicle emission attributes. Multivariate receptor modeling, such as Positive Matrix Factorization (PMF), can also be used to apportion chemical and/or size distribution data to the different source contributions. This has been used to identify NEE from atmospheric measurements,^29−31^ but studies (discussed in Section 3.1) have had limited success in separating individual NEE components and are likely to be location specific.

### 2.1 Non-Exhaust Emission Sources

#### 2.1.1 Brake Wear

**Figure 1:**
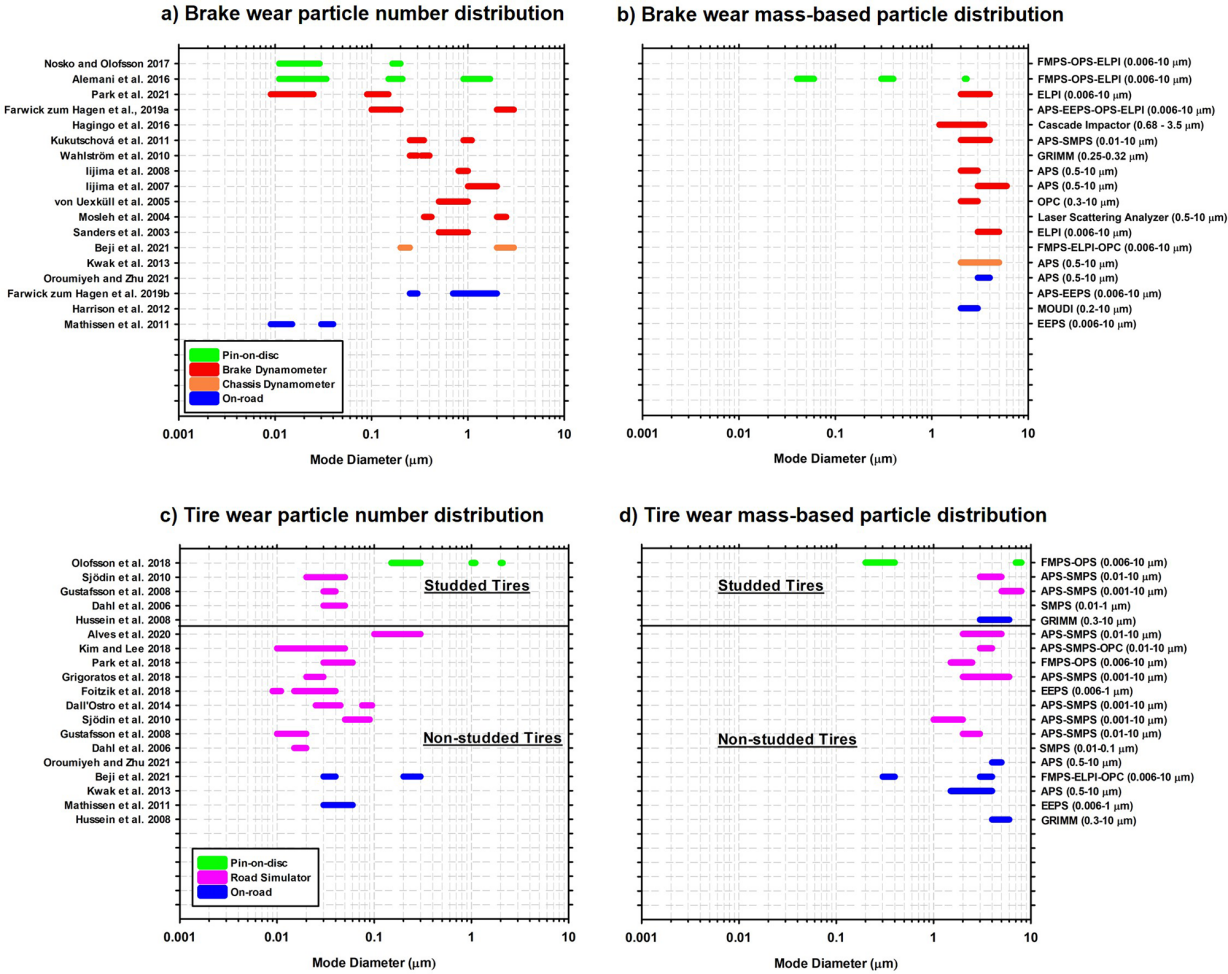
Overview of reported mode diameters of size distributions based on different measurement methods for (a) brake wear particle number distribution and (b) brake wear mass-based particle size distribution (c) tire wear particle number distribution, and (d) tire wear mass-based particle size distribution. Numbers in the parentheses show the detection size range of the measurement instruments: engine exhaust particle sizer (EEPS), aerodynamic particle sizer (APS), electrical low pressure impactor (ELPI), fast mobility particle sizer (FMPS), optical particle counter (OPC), laser scattering analyzer, optical particle sizer (OPS), scanning mobility particle sizer (SMPS).

During a braking event, mechanical interaction between the brake pad and rotor produces brake wear particles (BWPs) of different sizes^32^ (Figure 1a,b). While mass-based BWP size distribution in the fine and ultrafine size ranges have been reported,^33^ the majority of studies have reported a unimodal mass-based BWP size distribution with mode diameters in the range of 1–10 μm.^24,34−38^Figure 1 illustrates greater variability, with a mode diameter number distribution from nanoscale to coarse size range. Reported number distributions are influenced by factors such as brake lining material^19,39^ and maintenance history.^23^ Moreover, brake temperature can affect the BWP size distribution above a critical brake temperature (140 °C < *T*_crit_ < 240 °C) when ultrafine BWPs are generated.^23,40−45^*T*_crit_ increases during multiple runs of laboratory analysis of BWPs, presumably due to differences in volatilization onset temperatures of brake wear organic materials.^42^ For instance, *T*_crit_ of 180 and 240 °C has been reported for brake pads with organic and inorganic binder contents, respectively.^40,44^

Laboratory measurement studies have characterized the elemental composition of brake components.^34,37,46−48^ The metallic content of brake pads is dominated by Ba, Cu, Fe, Mn, Ti, and Zn.^49,50^ Other elements, including Al, Ca, Cd, Cr, K, Mo, Ni, Pb, Sb, Si, Sn, and Zr, have also been reported.^37,51−54^ In addition, while rare-earth elements were previously reported to be predominantly associated with mineral dust,^55,56^ a group including Gd, Ho, Lu, Pr, and Tb have been recently found in brake pads.^57^ The total carbonaceous fraction of BWPs can be highly variable (5–76%) depending on the brake pad material, braking velocity, and brake temperature.^21,58,59^ Approximately 150 organic compounds have been identified including *n*-alkanes, *n*-alkenes, *n*-alkanols, glycerol compounds, phenolic compounds, and polycyclic aromatic hydrocarbons (PAHs).^21^ Concentrations are higher during light- than heavy-braking events, indicative of thermal degradation.^21,60^ For brake discs, Fe has been reported to be the most dominant element.^50^

Elements most frequently used and deemed to be the most specific as brake wear tracers for source apportionment of fine and coarse particles are Ba, Cu, Sb, and Sn.^34,61−64^ Although others (e.g., As, Cr, Fe, Mn, Mo, Sr, Ti, Zn, and Zr have also been recommended^11,65−67^), many have been associated with other emission sources. For instance, Zn, Fe, Ti, Mn, and Sr can be found in tire wear, industrial emissions, and mineral dust.^11,56,68−70^

#### 2.1.2 Tire Wear

Variables influencing tire wear include tire characteristics (e.g., composition, construction, studs), road surface characteristics and vehicle operation/characteristics (e.g., speed, cornering, weight, power).^71^ Composition is also influenced by heat generated and the incorporation of other particles, most notably road surface material.^72−74^ Kreider et al. developed a nomenclature describing whether particles were generated from the original tread (TP), laboratory-generated tire wear particles (WP) or on-road collected particles (RP).^73^ The abbreviation TRWP (tire- and road wear particles) also encompasses the combination of these sources from a range of urban environments.^75^ A broad size range (10 nm to 10 μm) of laboratory generated TRWP has been measured (Figure 1c,d),^76−82^ with a unimodal mass distribution in the 1–7 μm range and number size distribution of 10–200 nm. On-road TRWP sampling and measurements on instrumented cars have adopted various approaches.^83−85^ Studies based on the TRAKER method report that particles sampled behind the front wheel of a car have a size distribution peaking at about 2–3 μm, with a high content of crustal elements, indicating a high contribution from road wear and dust.^38,86^ Beji et al, using electrical low pressure impactor devices, measured a bimodal size distribution (<0.03 μm and 0.05–0.30 μm by number; 0.1–0.6 μm and 1.0–15 μm by mass) when sampling from the front tire.^25^ Studded tires, used in cold climates to improve grip, generate ultrafine particles (<100 nm) (Figure 1c,d), that is speculated to originate from evaporation and subsequent condensation of softening oils in the rubber mix.^87^

Only 1% of tire wear is estimated to be released into the PM_10_ fraction,^73^ with varying contribution to atmospheric PM_10_: <1% using tire tread polymers as tracers,^75^ 0.1–3.9%^81^ and 1.0–7.5%^88^ using source receptor techniques, and 3–4% using simultaneous measurements of mass behind the front wheel and in the surrounding atmosphere.^38^ Few estimates of the contribution of tire wear particles to airborne concentrations exist, but available data indicate for the PM_2.5_ fraction: 0.1–0.68% of mass in London, Tokyo and Los Angeles using tire tread polymers^74^ and 4–7% using the mass based technique.^38^

Tires typically constitute rubbers/elastomers, fillers, processing oils, additives, reinforcements, and vulcanization agents, which vary depending on end use.^89^ A truck tire contains 80% natural rubber, whereas passenger car tires contain only 15%.^90^ Some of these can be used as markers for tire wear in the environment. Examples include components used in the vulcanization process, such as 2-(4-morpholinyl)benzothiazole^91^ and Zn,^92^ or those originating from thermal decomposition of tire tread polymers^93^ such as styrene, isoprene, dipentene, butadiene, vinylcyclohexene, and benzothiazole. The latter is used in the ISO standard for determination of TRWP.^94^ Compared to TP, TRWP are enriched in metals from brake linings and pavement materials and contain a lower concentration of polymers.^72,73^ Zn is a notable exception, which is enriched in TP relative to TRWP, despite being emitted by other sources such as industrial processes^70^ and brake pads.^50^

#### 2.1.3 Road Wear

Road surfaces are predominantly asphalt mixtures of ballast rock aggregates bound together with a bituminous binder, with several additives to improve durability such as fibers, resins, filler mineral powder, and polymers. In countries where studded tires are used, pavements have a high content of coarse wear-resistant rock aggregates in stone mastic asphalts. Smaller, less durable rock aggregates in less dense constructions can be used where studded tires are not common. The geographical availability of high-quality rocks also determines what is economically and environmentally feasible to use.

As vehicles tires move over the road surface, the interaction generates wear of both materials. Road wear particles (RWPs) are dominated by aggregate rock minerals that normally cover over 90% of the surface. Size distribution starts at ∼0.2 μm but particles are mainly in the coarse mode. Tests using road simulators, with and without studded tires, report a mean mass size distribution peaking at 6–8 μm^95,96^ and a bimodal appearance with mass peaks at 2–3 and 7–8 μm.^97^

Since most of wear dust originates from local crustal rocks, specific tracers are difficult to define, except in controlled studies where rocks with traceable composition are used.^98^ Abundant elements include Si, Al, Ca, K, Fe, and Ti. Size separated analyses suggest that these mineral related elements are abundant above ∼1 μm. Elements such as S and Cl, attributed to the binder matrix (bitumen), are more abundant below 1 μm,^99^ although these could also be related to tire wear. The bitumen of asphalt pavements contains a mixture of high molecular weight organic compounds. Approximately 5% of total suspended particles (TSP) in roadside samples in Denmark were bitumen particles.^100^

#### 2.1.4 Resuspension

Road dust consists of particles present on the road surface, generated by traffic or transported and deposited from near or long-range sources. Brake wear particles, RWP and TRWP originating from road and traffic, are major components of road dust,^101,102^ but winter traction sanding, building sites, wind-blown dust from bare soils, and dust dragged in by traffic from connecting unbound roads can be strong local sources. Source heterogeneity is reflected in the chemical composition (a mix of minerals, metals, and organic compounds) often occurring as aggregates with a high temporal and spatial variability. The RD10 (road dust smaller than 10 μm) fraction sampled in Oporto, Portugal, contained 11% organic carbon (OC) and 5% elemental carbon (EC).^103^ Metal oxides accounted for 30% and 73% in samples from asphalt and cobbled roads, respectively.^103^ The fraction contained hundreds of organic compounds of which plasticizers were the most abundant. Traffic related elements, such as Fe, Zn, Cu, Ba, and Sb are normally enriched in road dust in urban areas.^104^

The resuspension (or emission) of road dust is a complex process, dependent on road dust load,^105^ road surface macro texture,^106^ and humidity,^107^ traffic density, composition, and vehicle speed. Road dust load is a function of source strengths and the surfaces’ ability to contain dust under prevailing meteorological and traffic conditions and varies highly in time and space. A high macro texture can store more road dust but will make it less available for resuspension and thereby reduce emissions.^108^ Resuspension of dust is negligible when the road surface is moist and after rain events, and mobile dust load recovers faster in warm, dry climates compared to more humid ones.^109^ Water can also react with road dust, causing a cemented dust load in the texture that needs high suspension forces to be resuspended.

Resuspension of road dust is a strong PM source, especially in dry regions and where studded tires and winter maintenance (e.g., use of traction sand) are used. In Delhi, India, resuspension is estimated to account for 79% of PM^110^ compared with ∼38% of the traffic increment of coarse particles above the urban background, at a curbside site on a congested central London street.^92^ In Nordic regions, road dust accumulates during winter when sources are strong and the road surface is humid or frozen, causing a typical resuspension PM_10_ peak in early spring.^105,111,112^ Cross-street sampling demonstrates high variability with low loads in wheel tracks and high loads between/on curbs, implying differing resuspension potential depending on whether vehicles keep to the wheel tracks.^105^ PMF modeling estimates the composition of RD10 in Paris streets to be equal percentages of road wear, brake wear, and carbonaceous dust.^55^

### 2.2 Emission Factors

Nonexhaust emission factors (EFs) predict at wider geographical and temporal resolutions than can be achieved by measurements alone. They are typically presented as mg·km^–1^·veh^–1^ for different vehicle classes for the purpose of national atmospheric emission inventory reporting and can cover a range of driving styles. While distance-based EFs can account for speed and meteorological conditions (e.g., by applying correction factors), they are not dynamic and therefore constrained in providing very localized emission predictions, such as in areas with high levels of acceleration/braking.

#### 2.2.1 Brake and Tire Wear

**Figure 2:**
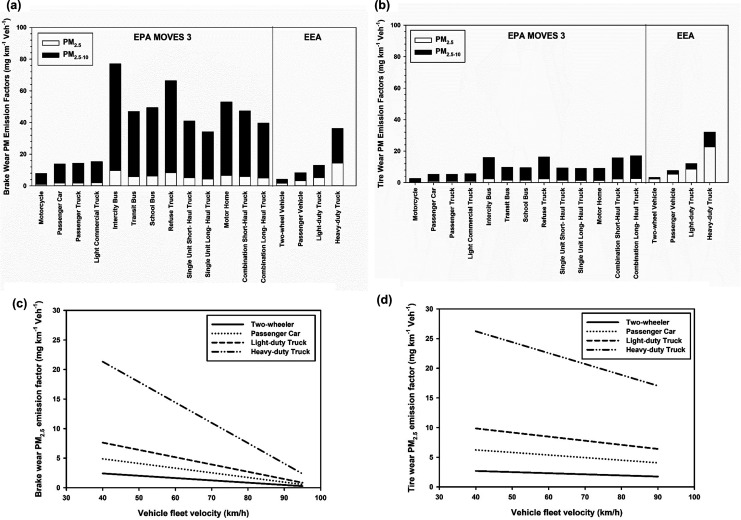
Effect of fleet velocity and weight on brake and tire wear PM emission factors (EFs) estimated by United States Environmental Protection Agency (USEPA) and European Monitoring and Evaluation Programme (EMEP)/European Environment Agency (EEA). Effect of weight on (a) brake and (b) tire wear PM EFs. Effect of vehicle velocity on PM_2.5_ EFs of (c) brake and (d) tire wear particles. EMEP/EEA brake wear PM EFs estimated for vehicle speed of 60 km/h EMEP/EEA heavy-duty truck brake wear PM EFs estimated for a half full truck with four axles.

Variability in estimated brake and tire wear PM EFs derived from different sampling approaches has called for updated emission inventories, taking into account various factors (e.g., braking activity, average deceleration rate, vehicle weight) based on results of multiple studies. The UK NAEI reported an average brake and tire wear PM_2.5_ EF of 3 and 5 mg·km^–1^·veh^–1^, respectively, and brake and tire wear PM_10_ EFs to be 7 mg·km^–1^·veh^–1^ (NAEI, 2018). Emission factors provided by the United States Environmental Protection Agency (USEPA), under the Motor Vehicle Emission Simulator (MOVES) program (Figure 2),^113^ for brake wear, PM_2.5_ and PM_10_ range from 1.0 to 9.6 and 7.8–77.0 mg·km^–1^·veh^–1^, respectively, and for tire wear PM_2.5_ and PM_10_ range from 0.4 to 2.6 and 2.7–17.1 mg·km^–1^·veh^–1^, respectively. Using the MOVES model, intercity bus and refuse trucks have the highest brake wear PM_2.5_ and PM_10_ EFs (Figure 2), exceeding those heavier vehicles (e.g., combination trucks), demonstrating the influence of frequent vehicle braking activity.

The European Monitoring and Evaluation Programme (EMEP)/European Environment Agency (EEA) proposed the most comprehensive method for estimating brake and tire wear, PM_2.5_ and PM_10_ EFs, based on TSP concentration and incorporating vehicle weight and velocity.^114^ For a similar vehicle category, EMEP/EEA reported higher brake wear PM_2.5_ EFs, but lower PM_10_ EFs (Figure 2) compared to the USEPA data, and higher tire wear PM_2.5_ and PM_10_ EFs than USEPA for any given vehicle class. This is likely due to the MOVES model estimating EFs from a limited number of older studies, assuming brake wear PM_10_ EFs to be ∼8 times higher than PM_2.5_,^19,113^ and using a tire wear PM_10_/PM_2.5_ ratio of 6.7 (recent studies have shown a tire wear PM_10_/PM_2.5_ ratio of 2.0–2.5).^77,113^ Incorporating the impact of vehicle velocity (Figure 2b,c), EMEP/EEA proposed negative linear relationships between the vehicle fleet velocity and brake and tire wear EFs,^114^ which have value for identifying brake and tire wear hotspots in urban environments.

#### 2.2.2 Road Wear

Among NEE, road wear is the least investigated, partly because direct emissions are difficult to separate from resuspended road dust. Using EMEP/EEA inventories and models, PM_10_ road wear direct EFs for passenger cars range from 5–10 mg·km^–1^·veh^–1^.^114,115^ A summary of five studies from road simulators, on-road measurements, and emission inventories, generated median EFs for light duty vehicles (LDV) of 7.75 mg·km^–1^·veh^–1^ and for heavy duty vehicles (HDV) 33.5 mg·km^–1^·veh^–1^.^9^ In countries where studded tires are used the road wear EF is harder to separate from resuspension, but has been estimated as 200–400 mg·km^–1^·veh^–1^.^99^

#### 2.2.3 Resuspension

PM_10_ EFs for resuspension on paved roads have been estimated using derivations from dust load, mobile measurements and vertical profile methods. Factors range from a few to ∼1000 mg·km^–1^·veh^–1^ for LDV^102^ and 150–8000 mg·km^–1^·veh^–1^ for HDV^116^ (using USEPA method based on silt loading and vertical profile of dust loading). PM_10_ resuspension was between 13 and 32 mg·km^–1^·veh^–1^ for 10 sites in Milan, Italy.^117^ A CO_2_ dilution approach determined average vehicle fleet resuspension EFs for dry (4–11 mg·km^–1^·veh^–1^) and moist/wet road surface conditions (2–7 mg·km^–1^·veh^–1^) on a congested London road, UK.^26^ The high variability is a result of geographical differences and possibly sampling, measuring and emission factor estimation methods. Modeling resuspension approaches include simple models using silt load in combination with mean traffic vehicle weight as the main influencing parameters,^118^ whereas more recent approaches take into account other factors (e.g., surface humidity and texture properties).^119^

## 3 Exposure Assessment of Non-Exhaust Particulate Matter

Exposure assessment of nonexhaust PM and sources is an emerging discipline. Estimation of airborne concentrations has used elemental tracers,^120^ but these are subject to interferences as no element is a wholly specific source tracer. Organic compounds have been used as tracers of tire wear,^121^ and pyrolysis/gas chromatography to quantify rubber.^122^ Element combinations may also be used in receptor modeling methods to identify factors consistent with nonexhaust sources.^123^ However, high levels of uncertainty surround all measurement methods. Common approaches for short- and long-term epidemiological studies include collection of monitoring data (from central stations, in-cabin vehicles, personal monitors), and use of high-resolution model predictions (from dispersion/chemical transport and land use regression (LUR)). Compared to the majority of studies on single air pollutant exposure (e.g., PM_2.5_ and gaseous pollutants), assessing nonexhaust exposure is more complex, requiring precise apportionment of PM mixtures for nonexhaust sources and accurate characterization of exposure for individual participants.

### 3.1 Methods for Assessing Short-Term Exposure to Nonexhaust Sources and Constituents

Short-term studies of associations between hospital admissions, emergency department visits and mortality, and exposure to daily PM constituents and sources have typically used data (e.g trace metals, EC and OC) from one central monitoring station per city to conduct source apportionment analyses.^124−132^ Strengths include long-term continuous data (usually >4 years) of high quality, allowing robust source classifications and estimations generated by a source apportionment model. The PMF model has been the most used given its flexibility to generate source contribution estimates without relying on a priori knowledge of source chemical profiles. Methods such as chemical mass balance (CMB),^123,130^ multilinear engine 2,^132^ and a hybrid model with CMB and non-negative factor analysis,^131^ have also been applied.

Many exposure studies have captured road dust as an indicator of NEE sources and linked it to epidemiological analyses. However, further separation of individual emissions has not been attainable from the PMF model, with only one exception, a study in Barcelona where the brake wear factor (Fe–Cu–Sb-rich particles) was disentangled from the mineral and road dust combinations in PM_10_ and PM_2.5_.^132^ One possible reason behind the difficulty in separating NEE source factors is that, because of the health relevance and data availability, many studies focus on exposure to constituents of PM_2.5_ rather than those in coarse particles (PM_2.5–10_), which form a significant part of NEE PM mass. Furthermore, depending on the representation of city monitoring sites, studies may not sufficiently capture near-roadway traffic-related sources, but rather indicate urban background mixtures. Lastly, many sources of NEE are spatially and temporally correlated.^123^

Directly measuring PM exposure in vehicles, or use of personal monitors may better capture specific NEE sources for epidemiological studies. A U.S. study measuring in-cabin exposure of patrol police officers to PM_2.5_ constituents identified two NEE sources indicating automotive steel wear (Ti–Cr–Fe particles) and speed-changing traffic (dominated by aldehydes–S–Cu).^133^ In the Atlanta Commuters Exposure (ACE) study, in-vehicle exposure to NEE were characterized by Ba, Fe, Mn, and S suggesting a strong contribution from brake wear.^123^ In a study in Hong Kong using personal exposure data and a PMF model, dust-related pollution was separated into crustal/road dust and a nonexhaust traffic-related fraction.^134^ It should be noted that, unlike ambient monitoring data, in-vehicle and personal exposure data are not geographically fixed. Therefore, these analyses rely on the assumption that the sources are the same across the driving/walking routes, which may complicate the interpretation of the source identification results.

### 3.2 Methods for Assessing Long-Term Exposure to Nonexhaust Sources and Constituents

Approaches taken to estimate long-term exposure to NEE sources and constituents include dispersion/chemistry-transport or LUR models. These are typically useful in characterizing intraurban contrasts at a small scale, such as the dispersion pattern of local traffic air pollution in large population studies with high spatial resolution.

Two popular dispersion models with capability to predict NEE include the UK KCLurban model developed for the Greater London area^135^ and a Swedish dispersion model primarily used in Stockholm, Umea and Gothenburg.^136^ These systems incorporate a detailed local emission inventory allowing apportionment of different local sources, such as the differentiation of exhaust and NEE from road traffic. Since the contribution of NEE in PM_2.5_ versus PM_10_ was empirically determined using field monitoring campaign data (in the Swedish model, 20–30% of PM_10_ was PM_2.5_), the uncertainty of the EFs is potentially larger for nonexhaust PM_2.5_ than total fine particles. To evaluate this uncertainty for PM_2.5_ and PM_10_ brake wear, tire wear, and resuspension, source apportionment estimates have been compared against observations from urban monitoring sites or field campaigns in a street canyon.^27,112,119^ Both models provide annual average estimations for nonexhaust PM_2.5_ and PM_10_ for many years on a regular 20 × 20 m (UK model) or 50 × 50 m (Sweden model) grid. This distinguishes exposures for a sizable population within a city, although several limitations remain. First, exhaust and nonexhaust PM exposures are highly correlated in their spatial dispersion due to their common generation presenting difficulties for discerning independent health effects of the individual sources. Second, model uncertainty that implies spatial agreement of annual mean prediction and observation data in long-term exposure studies presents challenges due to the small number of validation sites. Validation studies that incorporate additional monitoring data from field campaigns are therefore needed. Lastly, the UK and Sweden models are unique in terms of their detailed local emission inventory to apportioning exhaust and nonexhaust sources, and generalization to other areas with imperfect emission inventories is challenging.

For several cohort studies in California,, trace metals in PM_2.5_ (including Fe and Zn as indicators of NEE) have been estimated by a chemistry-transport model.^137,138^ Compared to dispersion models in Europe, these provide daily predictions over many years that are useful for both short- and long-term studies.^139^ While the model has performed moderately well for Fe and Zn across individual sites across cities, the coarse spatial resolution (4 × 4 km) prohibits investigation of fine-scale variations of traffic-related exposure.

LUR models are commonly used to assess long-term air pollution exposure,^140^ predicting spatial variations of trace metals in PM as markers of different sources in North America,^141−148^ Europe,^149,150^ Australia,^151^ and East Asia.^152,153^ These are empirically based on the relationship between measured air pollution and a number of geographical predictor variables, and estimate exposures at individual residential locations. Due to limited numbers of regular monitors for PM composition in a city, dedicated filter-based monitoring networks consisting of spatially dense (20–150 sites per city), but temporally sparse (7–14 sampling days in 2–3 seasons of a year) sites are often established to capture traffic-related PM sources and spatial dispersion. In this way LUR models are typically developed for annual or seasonal average predictions. Brake/tire wear PM composition markers have been determined either based on a priori knowledge from former studies (e.g., Cu, Fe, Zn in the ESCAPE (European Cohort Study for Air Pollution Effects)^150^ and MESA Air (Multi-Ethnic Study of Atherosclerosis and Air Pollution)^148^), or associations between trace metals in PM and traffic variables within small distance buffers (e.g., Ti, Cu, Fe in a New York city study).^141^ Overall, studies have shown that LUR models perform moderately well in explaining within-city variability of nonexhaust related trace metals (e.g., Cu, Fe, Ti, Ba, Zn) in PM_1_ (particulate matter less than 1 μm in diameter) PM_2.5_ and PM_10_ although less so than models for air pollutants that represent exhaust emissions.^154,155^ Potential challenges include imperfect study design, predictor variables, and modeling algorithms. For example, traffic-related predictor variables used in LUR models (e.g., road length, traffic volume, and near-road distance) cannot effectively disentangle exhaust and nonexhaust PM composition, preventing attempts to attribute health effects to separate sources. Predictor variables that reflect activities to produce/reduce brake and tire abrasion (e.g., road intersections, traffic speed) and road dust resuspension (e.g., use of studded tires, street cleaning, maintenance activities) are essential to improve nonexhaust LUR models. Recent studies have shown that there may also be value in incorporating machine-learning approaches to further improve model performance, since the dispersion pattern of NEE could be more local and highly nonlinear than exhaust pollutants.^149,156^ It is noteworthy that trace metals such as Cu, Fe, and Zn may not exclusively represent vehicle sources, with LUR models in Europe and Canada reflecting additional emissions released by metallurgical industries.^147,149^ Two U.S. studies have taken a further step by combining a source apportionment model with a LUR model to address the spatial variations of PM mixtures instead of focusing on individual constituents.^144,145^ Although both studies identified a brake wear source across the sites, the predictor variables were too general to explain source variations to a sufficient extent.

## 4 Epidemiological Studies

Although it is postulated that chemical composition may better explain observed PM-related health effects than mass alone,^157^ epidemiological studies of specific chemical components of PM, including constituents of NEE, remain scarce, in part due to limitations in exposure assessment. Studies have not yielded consistent results, with chemical tracers for combustion derived PM (EC, black carbon, Cr, V, Ni) often being more predictive^120,158^ and as a consequence, calls have been made for further studies.^159,160^ Studies examining PM composition have focused on a large set of species from ions (SO_4_^2–^, NO_3_^–^) and carbonaceous components (EC, OC) to metals (e.g., Ni, V, Cu, Zn, Fe). By and large when examining health effects, individual components are considered as tracers for sources. For example, and as discussed previously, in terms of NEE, Cu is typically used as a tracer for brake wear, Zn for tire wear (although there are industrial sources),^149^ and Fe for resuspended road dust.^161^ Epidemiological studies using source profiles obtained from dimension reduction methods including PMF often do not resolve a specific NEE source, but rather a traffic (primarily EC) or metal profile.^162^

### 4.1 Short-Term Studies

Time series and case-crossover models are typically used in assessing short-term associations with mortality or morbidity as a function of exposure adjusted for time and time-varying meteorological factors (e.g., temperature, humidity). Effect estimates are reported as odds ratios (OR) or percent increases per 10 μg/m^3^, or per interquartile range (IQR) change in PM or PM species. NEE components are examined directly or accounted for as a proportion of the mass that modifies the PM association (e.g., 0.75% excess risk (ER) in the PM_2.5_ estimate when the proportion of mass is higher in Zn). When components are examined directly, the models are generally adjusted for PM mass, but not other closely associated pollutants and constituents.

A systematic review synthesizing 32 short-term studies (31 time series, 1 case-crossover) found statistically significant pooled ER of cardiovascular-related mortality (ER = 0.49%, 95% CI 0.03–0.96%) per IQR increase in PM_2.5_ Zn.^159^ It also reported significant heterogeneity in cardiovascular morbidity with PM_2.5_ Fe and Zn (Cochran’s *I*^2^ = 52% and 62%, respectively). A study of Canadian hospital admissions found increased cardiovascular events in men associated with Cu, Fe, and Zn exposures when increased sulfur concentrations were also present (e.g., Cu OR = 1.08, 95% CI 1.05–1.11 per 10 μg/m^3^).^163^

The few epidemiological studies to use receptor modeling to identify a NEE component have not separately identified various sources, but report a single component, often referred to as “road dust”, but probably also containing other NEE sources. In terms of source contributions, both total and cardiovascular mortality were associated with 2-day lag exposure to road dust (loading on Cu–Fe–Zn–S) in a study of deaths in Barcelona, Spain.^164^ Rich et al. found IQR increases in road dust (containing Cu, Fe, Zn) on the same day were associated with significantly increased ischemic heart disease hospitalizations (ER = 0.6%, 95% CI 0.1–1.1%).^124^ Although not statistically significant, most NEE-associated ER of congestive heart failure, ischemic heart disease and myocardial infarction on the same day and previous 4 and 7 days were positive. A panel study of an identified NEE source representing “speed-changing traffic and with engine emissions and brake wear” (dominated by aldehydes–S–Cu) showed a strong association with heart rate variability in nine healthy, nonsmoking male highway patrol troopers.^133^

The aforementioned systematic review did not find any NEE markers to have significant pooled associations with respiratory mortality or morbidity.^159^ However, in a recent study of 100 patients with chronic obstructive pulmonary disease in Shanghai, China, greater levels of Cu and Fe were associated with a reduction in forced expiratory volume in 1s (FEV_1_) and forced vital capacity (FVC), and Cu was also linked to reduced peak expiratory flow.^165^ Similarly, a source factor representing NEE (loading on Ba–Fe–Mn–S) was associated with decreased FEV_1_ (−0.84% 95% CI −2.27--0.58) and increased airway inflammation in commuters in Atlanta, GA.^123^ The ACE study showed Cu to be significantly associated with increased exhaled nitric oxide and decreased FVC in 60 young adults.^166^

### 4.2 Long-Term (Chronic) Studies

Long-term (often cohort) studies are typically conducted using Cox proportional hazards models. Effect estimates are reported as hazard or risk ratios (HR/RR), or a change in risk associated with 10 μg/m^3^ or IQR increase in PM or PM species. Yang et al. synthesized 11 cohort studies and found significant pooled associations of nonaccidental mortality per IQR increase in PM_2.5_ Zn (ER = 9.4%, 95% CI 6.33%–12.56%), as well as cardiovascular mortality per IQR increase in PM_2.5_ Fe and Zn.^159^ Significant heterogeneity in PM_2.5_ Fe and Cu and cardiovascular mortality was also found (*I*^2^ = 95% and 90%, respectively). The ESCAPE studies found an increased relative risk of cardiovascular mortality with PM_2.5_ Cu (RR = 1.005, 95% CI 1.001–1.009) and respiratory mortality with PM_10_ Zn (RR = 1.136, 95% CI 1.010–1.277) in England, UK.^161^ In a pooled analysis of the 8 ESCAPE cohorts, robust associations were found between all-cause mortality and linear regression modeled PM_2.5_ Cu, Fe, and Zn in single pollutant models, and when adjusted for PM_2.5_ mass.^167^ Cardiovascular and lung-cancer mortality showed similar RRs for these NEE components, but they did not remain statistically significant when adjusted for PM_2.5_ mass. In a population-based Canadian cohort, long-term exposure to PM_2.5_ Fe and Cu were consistently associated with increased cardiovascular mortality (Fe HR = 1.014, 95% CI 1.008–1.019; Cu HR = 1.006, 95% CI 1.001–1.012) and congestive heart failure (Fe HR = 1.031, 95% CI 1.024–1.038; Cu HR = 1.015, 95% CI 1.009–1.022).^168^

Fuertes et al. found that across seven European birth cohort studies, PM_10_ Zn was the only element independently associated with a higher risk of early life pneumonia (OR = 1.47, 95% CI 0.99–2.18).^169^ In a Dutch birth cohort, asthma symptoms were positively associated with PM_10_ Cu (1.06, 95% CI 1.00–1.12), as was decreased FEV_1_ (−2.3%, 95% CI −4.3--0.3%).^170^ PM_10_ Cu and Fe were associated with increased allergic sensitization, and PM_2.5_ Cu and Fe were associated with decreased FEV_1_.

A meta-analysis of 32 studies found a significant decrease in birth weight per IQR increase in PM_2.5_ Zn (pooled effect −7.5 g, 95% CI −10.0 g to 5.0 g).^171^ A UK study found elevated risk in preterm birth associated with nonexhaust PM_2.5_, modeled as brake/tire wear and resuspension of road dust (OR = 1.03, 95% CI 1.01–1.05).^172^ In multipollutant models, elevated PM_2.5_ Zn was associated with higher mean diffusivity (a marker of neurodevelopment linked to psychiatric and neurological disorders) in Danish children.^173^ Another study reported an increased OR of autistic disorder in children by age 6 years when mothers were exposed to elevated concentrations of Cu during pregnancy.^174^

In summary, short- and long-term studies of PM_2.5_ and PM_10_ elemental composition have been conducted, some focusing specifically on certain NEE tracer elements or source factors. There is consistency in the findings for PM Zn and road dust source factors being associated with acute and chronic cardiovascular outcomes, as well as birth outcomes. Several components of NEE showed significant associations with children’s respiratory health and neurodevelopment.

## 5 Toxicology/Mechanistic Data

The physicochemical properties of NEE indicate considerable potential to cause adverse biological responses if inhaled, either through activation of resident airway inflammatory cells, or via interactions with the underlying epithelium. However, compared with the extensive literature on combustion particle toxicity, relatively few studies have directly addressed the hazards associated with NEE exposure. Those that have, mostly focus on the capacity of NEE-derived PM to induce oxidative stress as a trigger for acute inflammation and cellular injury, with a focus on metals and organic species derived from mechanical wear.^175^ These species can catalyze the formation of ROS, either directly, through catalysis of the Fenton and Haber-Weiss reaction (Fe, Cu, Mn, etc.)^176^ or indirectly (in the case of Zn) by interfering with intracellular signaling through inhibition of phosphatases,^177^ through the generation of reactive electrophiles and ROS through the induction of xenobiotic pathways (organic components),^178^ or disrupting mitochondrial function.^179^

### 5.1 NEE in Roadside PM

The largest body of work that indirectly examined NEE toxicity compared roadside (enriched with NEE source contributions) versus urban background/rural PM, relative to source-specific reference PM standards (typically primary diesel exhaust particles (DEP)). Researchers have generally attempted to dissect out contributions of individual components of NEE through correlating the range of toxicological end points to imperfect chemical tracers (usually metals/metalloids: Zn for TWP; Cu, Ba, Sb, and Sn for BWP; Fe, Cu, and Si for RWP; versus Ni, Cr, and V for oil combustion). As many of these components are highly correlated within the panels of PM tested, this method has inherent limitations. However, several studies augmented this observational approach through inhibition of component-specific biological pathways (metal handling,^180,181^ aryl hydrocarbon receptor mediated xenobiotic metabolism,^180^ toll-receptor mediated responses to PM associated biological components^182^ or PM fractionation^183^).

Several in vivo animal studies examined the NEE contribution to RWP toxicity. Gerlofs-Nijland et al. comparing the effects of coarse (PM_2.5–10_) versus fine (PM_0.18–2.5_ accumulation mode) RWP collected at various European sites, in rats following pulmonary instillation.^184^ The results were complex but did show that PM from traffic heavy sites induced pulmonary inflammation and raised circulating concentrations of blood fibrinogen (an acute phase response protein involved in coagulation). These effects were correlated with Cu, B, and Zn concentrations (reflective of mixed NEE sources), but not PAH content. In many cases, effects were only seen at the highest exposure levels (∼2.5 mg/rat) and the authors noted the actions of certain markers could have arisen from sources such as wood-smoke. Happo and colleagues (2010) investigated different sized urban PM samples (PM_10–2.5_, PM_2.5–1_, PM_1–0.2_, PM_0.2_) from Helsinki, Finland,^185^ collected at different seasons to capture spring-time increases in PM_10–2.5_, caused by resuspension of winter road dust. Selected elements were used as indicators of NEE (road dust: Ca, Fe, Al, Mn; tire and brake: Cu, Zn, Fe). Instillation in mice (∼0.25 mg/mouse) resulted in pulmonary inflammation, with NEE-enriched samples inducing the most pronounced response, especially with coarse mode PM containing elevated endotoxin (a bacterial product). These samples also exhibited relatively high EC/OC ratios (indicative of combustion-derived PM), making it challenging to separate the effects of exhaust and NEE. Nonetheless, the authors concluded traffic-derived PM from mixed NEE sources was the key driver of the inflammatory responses. Kreider and colleagues (2012) carried out a subacute inhalation study of mixed TWP and RWP in rats.^186^ Lower doses were tested (10–100 μg/m^3^, 6 h/day for 28 days), with no evidence of enhanced mortality or gross pathology effects. Occasionally foci of lung pathology were observed with high exposures (100 μg/m^3^), but were unaccompanied by markers of lung or systemic inflammation, oxidative stress, or thrombosis. The authors concluded a no-observed-adverse-effect level of 112 μg/m^3^ - well above environmental levels, for this animal model (the potential human implications are discussed in Kreider et al.^187^). The study also contained unpublished instillation data in the discussion that compared different types of PM. PM from tire-brake wear did not significantly increase pulmonary inflammation, whereas DEP or crystalline silica did. The authors therefore suggested that, despite TWP and BWP presenting a significant fraction of PM mass, they did not contribute significantly to ambient PM toxicity.

### 5.2 Toxicity of Brake Wear

Increasingly, in vitro and in vivo evidence suggests that metals within BWP (specifically those capable of catalyzing ROS production), are important contributors of toxicity. This was first addressed by Gasser et al. using freshly generated BWP and an alveolar epithelial cell line (A549).^188^ Exposure was carried out at the air–liquid interface; a more physiological model than conventional submerged cell culture. A range of driving and braking behaviors were explored, with the number of repetitions and more abrupt braking modes associating with increased particle mass, number, EC/OC, and metal (Fe, Cu, and Mn) content. While little evidence of overt cytotoxicity was observed, reduced tightness of the cellular layer, increased oxidative stress (especially with particles produced by full-stop braking) and heightened release of the cytokine IL-8 were observed. The reduced cellular tightness was correlated with particulate Fe, Cu, and Mn content, while inflammation correlated with OC. These associations were, however, based on a limited number of observations and concentrations of Fe, Cu, and Mn were highly correlated, preventing assessment of their relative contributions to the biological responses.

Focusing on the role of Cu in BWP toxicity, Figliuzzi et al. exposed A549 cells to PM_2.5_ emitted from four different pad/disc combinations.^189^ Exposures to 1–500 μg/mL PM (equivalent to 0.0018–70 μg/mL Cu) caused dose-dependent increases in ROS production. Reductions in cell viability were reported at the highest doses (50–500 μg/mL), but not for BWP with mid and low Cu content. Highly Cu-enriched particles disrupted mitochondrial membrane integrity and increased the number of apoptotic cells. Contrastingly, and in agreement with the observations of Gasser et al.,^188^ up-regulation of genes relating to oxidative stress and inflammatory responses did not correlate simplistically with particulate Cu content. Barosova et al. used a more sophisticated 3D coculture consisting of A549, monocyte-derived macrophages and dendritic cells challenged with specific size fractions (0.25–1, 1–2, and 2–4 μm) of BWP derived from pad composites of low to moderate metal content.^190^ The low metal composite-derived PM elicited no toxicity with any size fraction, whereas the high metallic composite increased IL-8, albeit with no clear dose response, or variation between the collected PM fractions. There were no alterations in intracellular glutathione metabolism, or indications of cell injury. While these findings have been cited as providing evidence of brake dust metal toxicity, it should be noted that the experimental work did not include an analysis of organic chemical species in the PM fractions, an examination of metal handling pathways, or employ selective metal chelators to confirm findings.

Selley et al. compared the toxicity of a composite BWP sample from European buses and trucks with that of a standard reference material DEP.^181^ Samples varied considerably in their metal/metalloid content, especially for those capable of producing ROS (Fe, Cu, Mn, Mo), indirectly inducing oxidative stress (Zn, Ca) or distinguishing BWP (Ba, Sb, Sn). The DEP contained very little metal, though concentrations of V, As and Ti were broadly equivalent in the two samples. Despite this compositional contrast, both particles induced mitochondrial depolarization, decreased phagocytic capacity, and heightened IL-8 secretion in monocyte-derived macrophages at equivalent doses (4–25 μg/mL). As phagocytosis is a primary mechanism by which macrophages protect the airway from pathogens, the authors hypothesized that both particles could enhance susceptibility to infection. While the similarity in response did not support metals being key drivers of BWP toxicity, responses were inhibited by the metal chelator desferroxamine for both particles, suggesting that even low concentrations of metals can instigate toxicological effects. This study also highlighted the danger of equating the biological/cellular dose of metals to the measured PM content, as only a subset of the PM-associated metals was shown to dose-dependently accumulate in macrophages.

**Figure 3:**
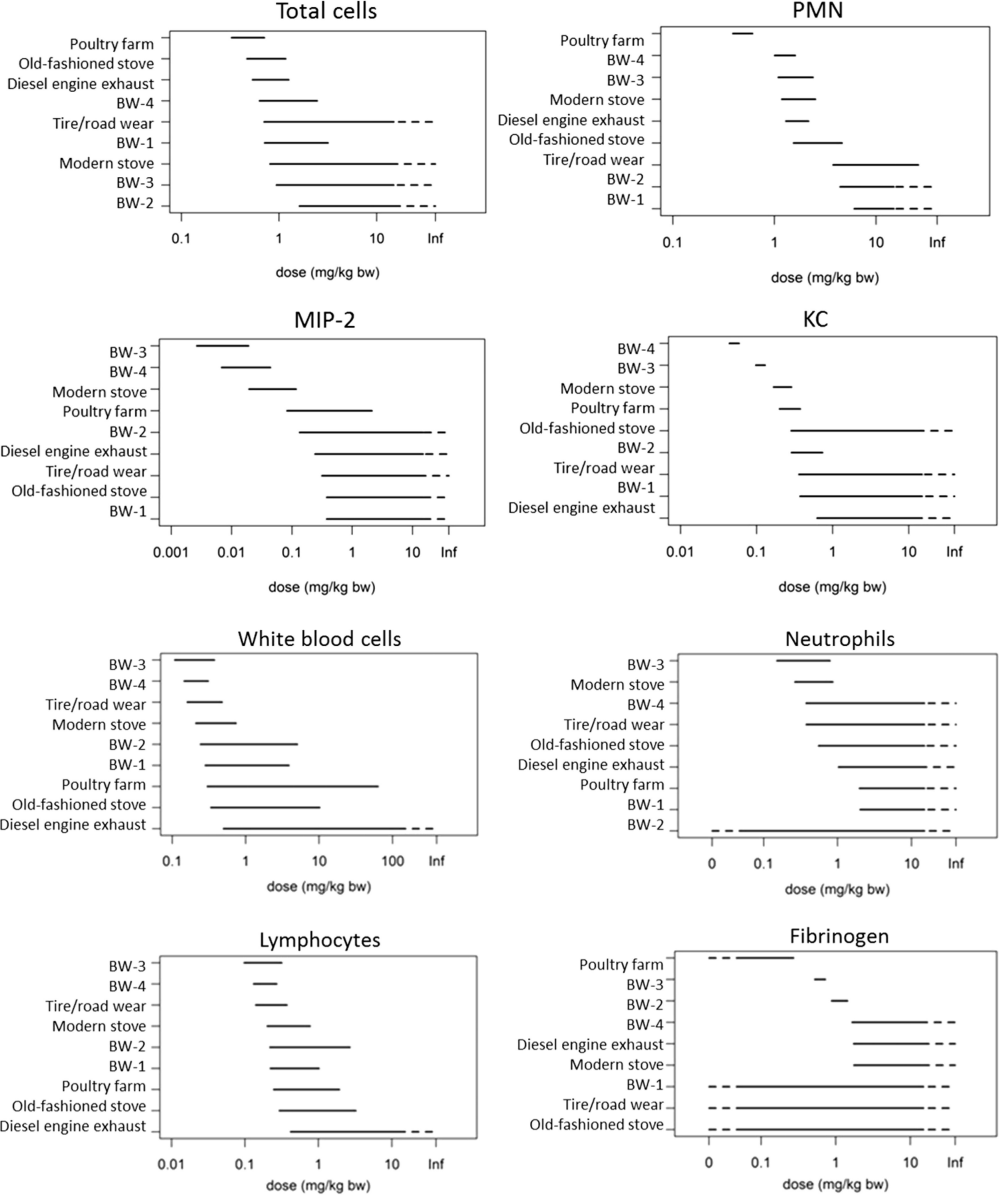
Relative toxicity of BWP compared to particulates from other sources following inhalation in mice. The further the line is to the left, the lower the dose at which it exerts an effect (i.e., the greater the toxicity) for that given parameter. In several assays, BWP had a greater effect than tire wear, DEP, and other PM. Equally, the relative toxicity varied considerably between the parameter being studied. Upper four panels are measures of pulmonary inflammation, lower four panels are markers of systemic inflammation. BW-1 = BW from low metallic pads but with some copper, BW-2 = BW from semimetallic pads with no copper, BW-3 = BW from organic brake pads, BW-4 = BW from organic and metallic hybrid pads, KC = keratinocyte-derived chemokine, MIP-2 = macrophage inflammatory protein, PMN = polymorphonuclear neutrophils. Gerlofs-Nijland, B. G. H. Bokkers, H. Sachse, J. J. E. Reijnders, M. Gustafsson, A. J. F. Boere, P. F. H. Fokkens, D. L. A. C. Leseman, K. Augsburg, and F. R. Cassee (2019) Inhalation toxicity profiles of particulate matter: a comparison between brake wear with other sources of emission, Inhalation Toxicology, 31:3, 89–98, 10.1080/08958378.2019.1606365 by Informa UK Limited, trading as Taylor & Francis.

Several studies have explored BWP toxicity in animal models. We will not, however, cover those that employed asbestos-containing BWP (reviewed by Poland and Duffin^191^). Gerlofs-Nijland et al. carried out a comprehensive comparative toxicology study in mice, investigating PM from four types of brake pads (low-metal, semimetal, organic, and semimetal–organic hybrid PM) and TRWP from laboratory simulators, as well as DEP, wood-smoke and PM collected in the vicinity of a poultry farm that was enriched with biological components.^192^ Inhalation (9 mg/m^3^ for up to 6 h) of the high organic and hybrid metal–organic BWP, but not the semimetallic BWP, induced lung inflammation and mild increases in blood leukocytes and fibrinogen. In some cases, the organic-rich BWP had greater inflammatory effect, above TWP, DEP, or wood smoke (Figure 3). While organic-rich brake-wear PM had more marked biological effects than the metallic-BWP, it should be noted that levels of Cu in the organic-PM was actually 4–6 times higher than the metallic brake pads. Furthermore, other pulmonary end points (additional cytotoxicity assays and oxidative stress by glutathione depletion) were unchanged by any of the PM types, and where effects were seen, they were induced by the highest dosing period (6 h exposure). No effects were observed following the shortest dosing period (1.5 h), which the authors suggested was representative of human exposures. This clearly demonstrates that PM with different source profiles elicit markedly different immune responses in vivo.

### 5.3 Toxicity of Tire and Road Wear

Studies of TWP toxicity remain scarce, and due to the methods used to generate the particles, often investigate mixed-source samples from the tire-pavement interface. PM_10_ generated through wear of studded tires with asphalt or granite mixtures has been shown to induce genotoxicity and mitochondrial depolarization in lung epithelial cells^193−195^ and pro-inflammatory cytokine secretion from macrophages.^193,196^ Compared with BWP, studies of TWP have explored a wider range of toxicological end points. Karlsson et al. performed proteomic profiling of primary human monocyte-derived macrophages exposed to 100 μg/mL PM_10_ from wear of studded tires against asphalt.^197^ The authors did not identify sufficient differentially expressed proteins to perform a statistical pathway analysis but noted that the exposure altered proteins required for inflammation, fibrosis, actin remodelling, and energy metabolism. As well as metals, organic compounds are postulated to mediate TWP toxicity. In a series of experiments with A549 cells, Gualtieri et al. demonstrated that the organic fractions of milled tires (mainly composed of isoprene polymers) induced cytotoxicity, DNA damage and cell cycle arrest following longer exposures (72 h) at doses >60 μg/mL.^198^ Subsequent experiments linked toxicity to ROS generation, inhibition of protective Hsp70 activity^199^ and redistribution of plasma membrane lipid microdomains.^200^

In vivo, three studies of responses to TWP exposure have been performed. Gottipolu et al. investigated two types of respirable TWP that leached Fe, Cu, Zn, and Al.^201^ Following pulmonary instillation in rats (∼1.5 mg), both PM types increased several markers of pulmonary inflammation, with TWP that contained more Cu and Fe exerting greater effects. Effects were seen at 24 h post exposure and returned to baseline by 1–4 weeks. Neither TWP affected cardiac enzymes. The authors demonstrated that nontire sources of soluble Zn and Cu exerted similar pulmonary responses, albeit the concentration tested was orders of magnitude higher than those leaching from the tires.

A similar approach by Mantecca et al. compared size-fractionated TWP (PM_10_ and PM_2.5_) where PAH concentrations were enriched in PM_2.5_.^202^ Both PM size fractions induced pulmonary inflammation after instillation into mice (10–200 μg) with greater effects observed with the smaller PM fractions (although responses were variable and often not dose-dependent). Only the high PM_2.5_ doses were associated with overt changes in lung histopathology. The authors suggest a different mechanism of action between the two particle fractions, with macrophages being heavily involved in responses to PM_10_ and cellular cytotoxicity being largely governed by PM_2.5_. The same group compared size fractionated tire particles and similarly sized urban PM collected in Milan, Italy.^203^ As before, the PM_2.5_ size fraction provoked an enhanced pulmonary response (inflammation, apoptosis and some signs of adverse histopathology), whereas PM_10_ TWP generally did not. Interestingly, the effects were comparable to, or even greater than urban PM in some assays. The authors calculated that an equivalent dose of PM_2.5_ in humans would be 8 h of 425 μg/m^3^; a very high level of exposure, but one that could be reached in an extreme pollution episode in some megacities (although only a small proportion of this would be expected to be derived from TWP). Gerlofs-Nijland et al. performed a comprehensive investigation of the acute toxicity of mixed tire and RWP to that of other sources (see Section 5.2 for details).^192^ TWP had a modest capacity to induce pulmonary inflammation, although the relative toxicity varied with end point, with PM from other sources (e.g., BWP, woodstove, particles collected near a poultry farm) often inducing greater responses.

### 5.4 Overview of Toxicological Studies

Studies show that NEE have the capacity to induce biological actions indicative of potential adverse health effects. In vitro studies have employed several methodological designs, dose ranges, and cell types, although monolayers of lung epithelial cells are the most frequently studied. Both BWP and TWP can exert cytotoxicity at high doses, through different biological mechanisms with inflammation and oxidative stress being the most studied. Several studies indicate that metals and PAHs are key components driving the toxicological actions observed, consistent with the wider literature on ambient PM. In vivo exposures of NEE in rodents are almost exclusively focused on pulmonary actions, with a handful of studies also examining blood biomarkers. As with in vitro studies, the biological pathways explored/identified are those already established for urban PM and exhaust PM, that is, oxidative stress, coagulation, and inflammation.

In summarizing this literature, we highlight several points. First, numerous studies use PM collected at roadside, and employ measurement of tracers (usually metals) to infer which effects may be linked to nonexhaust PM. However, the proportion of nonexhaust PM within these samples is often not clear and tracers used could originate from other sources. Second, while several studies have considered responses to different particle size fractions, greater attention has focused on coarse and fine PM in NEE, with few studies specifically addressing ultrafine particles. The greater relative surface area of the ultrafine particles, the degree of penetration into the lungs and their ability to translocate into the blood and organs could engender these particles with greater (and different forms of) toxicity. Indeed, there is increasing evidence that ultrafine particles can pass into the circulation and be carried to other organs of the body.^204^ Maher and co-workers used magnetic quantification and imaging techniques to demonstrate the presence of iron-rich particles (10–150 nm, also containing trace amounts of Pt, Ni, Co, and possibly Cu) in both the brain^205^ and the heart^206^ of cadavers from Mexico and the UK. It has been argued that the rounded morphologies and fused surface textures of these particles may suggest formation at high temperature, possibly from friction on braking. While it is not possible to conclusively prove that these particles have biological actions, cardiac cells containing these particles were found to have histopathological alterations in mitochondrial structure and higher levels of cellular prion protein, consistent with oxidative injury to this organelle. Third, few studies directly compare the biological effects of nonexhaust PM to other PM types. In vitro^207^ and in vivo^192^ studies have shown that some types of nonexhaust PM have greater effects than the same concentration of exhaust PM, but observations were not consistent across different studies or even end points within a given study. In contrast, Kreider et al. referred to unpublished data in rats showing that NEE had no significant effect on lung inflammation, whereas DEP increased the number of inflammatory cells in the lung.^186^ Finally, as is conventional in toxicological studies, higher dose ranges are employed to establish effects and thereafter address potential underlying mechanisms. Dose-dependent relationships between particle concentration and effect are found (commonly in in vitro studies, less so in vivo), although significant effects tend to be observed only at higher concentrations, and may not simplistically equate to effects at real world concentrations in humans. Focusing on the two prominent inhalation studies, one study found that short-term exposure to high concentrations (6 h at 9 mg/m^3^ PM) of brake wear PM induced modest lung inflammation and mild changes in indicators of systemic inflammation.^192^ The other study that used prolonged exposures of moderate doses (28 days at 0.1 mg/m^3^ PM) did not find a mixture of NEE types to have effects on any parameter studied.^186^

Overall, based on current research, several forms of NEE have the capacity to induce adverse effects in the lung. These effects are likely linked the ability of NEE to induce inflammation via the induction of oxidative stress, and other forms of biological dysfunction These studies should stimulate further research, specifically focusing on: (a) impacts other organ systems, (b) toxicity of the ultrafine fraction of NEE, (c) comparative toxicology with other PM sources within the urban environment, and across a range of models, reflective of disease vulnerabilities and (d) at realistic doses. There is also the need to further dissect out the molecular pathways governing adverse outcome pathways linked to components within NEE.

## 6 Future Scenarios and Mitigation of Non-Exhaust Emissions

### 6.1 Future Scenarios for Nonexhaust Emissions

**Figure 4:**
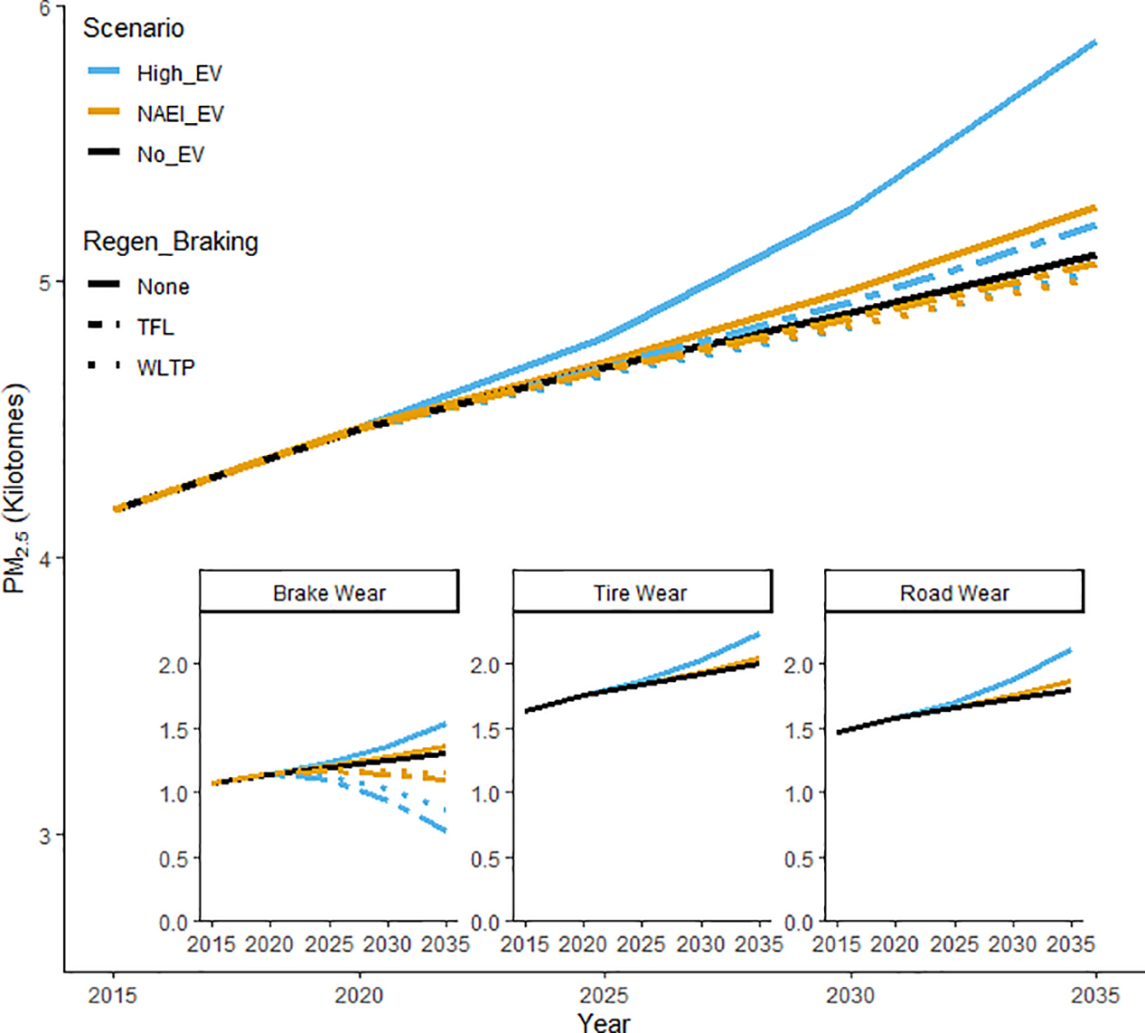
Projected PM_2.5_ brake, tire, and road wear emission estimates for passenger cars in the UK based on changes in DfT (2018) modeled traffic volume and battery electric vehicle uptake (vehicle mass, and regenerative braking). A vehicle mass EF regression approach^208^ was used to determine the impact of heavier EVs, while reductions from regenerative braking were calculated using brake force simulations for passenger vehicles under the TfL (dot dashed −65% reduction) and WLTP (small dots −88% reduction) drive cycles. Three scenarios have been considered: “NO_EV”: DfT reference (Scenario 1) traffic projections assuming no electrification of the vehicle fleet; “NAEI_EV”: DfT reference (Scenario 1) traffic projections + UK NAEI EV uptake; “High_EV”: DfT shift to zero emission vehicles (Scenario 7) + high uptake of EVs. DfT: Department for Transport; EF: emission factor; EV: electric vehicles; NAEI National Atmospheric Emissions Inventory; PM_2.5_: particulate matter less than 2.5 μm in diameter; TfL: Transport for London; WLTP: Worldwide Harmonized Light Vehicle Test Procedure

The magnitude of future NEE is uncertain due to the unquantified impact of increased vehicle weight, high torque, and regenerative braking associated with electric vehicles (EV),^208,209^ as well as the projected increase in EV mileage due to lower running costs and increased range per battery charge.^210^ This is illustrated graphically in Figure 4, in which brake, tire, and road wear emissions from passenger cars in the UK are forecast to 2035 under different scenarios based on the EMEP/EEA Guidebook EFs^114^ and vehicle mileage (e.g., activity data).^210^ As shown, the “NAEI EV”^211^ scenario (moderate uptake in EVs) and an assumption of no reductions from regenerative braking, would lead to a 3% increase in NEE and the “high EV”^210^ scenario to a 15% increase, compared to the “No EV” scenario. Regenerative braking would mitigate these effects (∼60–90% reduction) by a level dependent on the drive cycle used. These are comparable to an analysis by Hooftman et al., suggesting that regenerative braking reduces brake wear emissions by approximately 66% (despite the heavier mass of EVs),^212^ considering service time of brake linings from internal combustion engines and EVs in urban settings. Beddows and Harrison compared emissions of EV with those of near-equivalent petrol and diesel passenger cars, and assuming 90% regenerative braking on urban and rural road types, there was a small reduction in total nonexhaust particles for the EV despite their greater weight.^206^ However, regenerative braking does not offset the additional PM_10_ from heavier EVs on motorways.^206^

### 6.2 Mitigation of Nonexhaust Emissions

To reduce NEE, a range of legislative, traffic management, and scientific engineering measures are needed. Reductions in traffic volumes, vehicle speed/velocity, and aggressive driving styles will reduce brake, tire, and resuspension emissions. This could be facilitated by infrastructure design, including road alignment, optimization of road surface texture and traffic signal co-ordination.^213^ Trade-offs in the selection of options should, however, be considered. For instance, a low microtexture that decreases tire wear will also reduce friction and hence compromise safety. In contrast macro texture properties might reduce tire wear while also reducing noise and rolling resistance.^214^ Targeting tire/road wear and resuspension emissions is especially important as they increase with vehicle weight and as such, electrification of the fleet. Decreasing the mass of vehicles, especially that of EVs (e.g., by size, design, materials, Li-ion/energy-dense battery technology, smaller EV batteries) would also reduce several nonexhaust sources.^24,208,209^ The use of lighter materials (e.g., carbon composites) may, however, come at the cost of higher environmental impacts during the production phase and lower recyclability of materials.^215^ Introducing taxes on distance traveled and vehicle weight would reduce mileage and discourage heavier vehicles, respectively.^216^ Although these measures may be considered politically complex to implement, they would bring about wider benefits on exhaust/greenhouse gas emissions, road safety, and congestion.

#### 6.2.1 Brake Wear Mitigation

Several US states have set limits on the content of certain heavy metals, (e.g., Cu, Cr, Cd) and asbestos in brake pads has been banned in most regions of the world.^77^ Although these legislations could reduce heavy metals in brake material, they may not successfully reduce overall brake wear emissions if alternative materials have comparable wear properties. It is important to consider specific brake materials (e.g., titanium, aluminum, ceramic, or carbide coatings) with lower wear properties and reduced brake wear emissions. Tungsten carbide and carbon ceramic discs have been shown to reduce PM_10_ emissions by up to 70%,^217^ while results indicate potential for a European standard car disc brake system to reduce brake wear PM_10_ by 32–62%.^218^ Future legislation could facilitate the uptake of alternative brake wear materials through financial incentives. Technologies to catch brake wear particles at the source through filtering devices mounted on the brake disc are also under development.^219,220^ It is expected that brake materials will change dramatically in the future and keeping track of composition and tracers is crucial for apportionment studies and identification in environmental samples. Brake wear emissions and, importantly, the ultrafine fraction can also be mitigated by promoting smoother braking and less aggressive driving.^45,221^

#### 6.2.2 Tire Wear Mitigation

Tire materials and construction can be optimized toward lower wear but must be balanced against properties related to safety (friction) and environmental (noise, rolling resistance) aspects. Harder rubber might, for instance, reduce wear but decrease friction and increase noise. A tire wear test method to facilitate tire wear marking, enabling customers to choose a lower wearing type, is being investigated within industry^222^ and research initiatives (e.g., EU-project LEON-T^223^). While there is already a tread wear rate marking on some tires marketed in the U.S., this does not correlate well to mass loss.^77^ Alternative materials to rubber, a major source of microplastic pollutants, could also contribute to lower emissions. However, the driving forces behind the large effort to find alternatives to synthetic and natural rubber focus on (a) raw material production and transport to reduce greenhouse gas emissions, and (b) agricultural and recycling aspects rather than reducing the wear of the final product. There are also initiatives aimed at collecting TWP while driving.^224^ Road surface roughness/condition affect the wear of tires, thus efforts could incorporate adapting surface properties.^222^ The driver/car owner can reduce tire wear through driving behavior (e.g., lower acceleration, soft braking/steering maneuvers) and maintaining correct tire inflation pressure and wheel alignment.^225,226^

#### 6.2.3 Road Wear Mitigation

Road wear is a substantial part of NEE, yet it has not earned much attention outside the studded tire zone. In Sweden the amount of pavement eroded by studded tires was approximated to ∼110 000 tons each year, despite the development and use of very wear resistant surfaces since the 1970s.^227^ Measures to reduce the use of studded tires through charging schemes (Norway) and prohibition on certain streets (Sweden) have helped to reduce PM concentrations.^228^ Other measures focus on designing tires with fewer studs per wheel circumference, which produce less abrasion. Apart from wear resistance, the surface condition affects particle emissions. A study of unstudded tires on pavements in a load simulator concluded that a pavement surface in good condition had very low emissions, while damaged surfaces lead to considerably more.^95^ Compared to smoother pavements with finer aggregates used outside the studded tires zone, wear-resistant pavements are also characterized by higher noise emissions and rolling resistance. The potential impact of the latter on health and fuel consumption means that efforts to reduce them are higher on the mitigation agenda than wear emission reduction. Reducing aggregate size,^229^ use of alternative materials in the matrix,^230^ and adopting porous pavements^231^ are some of the measures that have been investigated to reduce noise. Using residual products in the matrix such as scrapped tires and incinerator slag offer an alternative environmental benefit, although current findings are mixed and evaluation of PM emissions is lacking.

#### 6.2.4 Resuspension Mitigation

Resuspension can be mitigated by reducing sources of road dust (Sections 6.2.1–6.2.3) and/or dust suspension. Measures to reduce suspension include reducing surface macro texture (to reduce dust accumulation and facilitate sweeping) and ensuring road surface maintenance to guard against cracks and other dust accumulating damage. In 2015, a European certification test, assessing the PM_10_/PM_2.5_ efficiency of road sweepers, was established.^232^ Road sweeping tests on real roads often show limited efficiency,^233,234^ explained by the failure of techniques to collect and hold small particles.^235^ Techniques can sometimes cause resuspension themselves through brushing without simultaneous dust suppression. Studies combining sweepers and washers^236^ or high vacuum sweepers equipped or combined with high-pressure washing (reducing dust load of sub-180 μm particles by up to 92%)^237,238^ have reported more promising results. Another well-tested measure is dust binding (or suppression), normally accomplished through spraying water or hygroscopic solutions (chloride salts, acetates, formates) over the road surface to keep it moist, thereby preventing suspension. Efficiency depends on agent used, dose, concentration, and application criteria, and ranges from a few percent to 70% reduction of resuspended PM_10_.^239^ The effect of dust binding is rather short-lived and dependent on traffic intensity, temperature, and humidity (i.e., parameters affecting how fast the road surface dries).^112,119^ A study in the warm and dry climate of southern Spain showed that a dust binding agent had very little effect, but street washing with high amounts of water was more effective.^55^ Porous pavements have also been shown to conceal dust, thereby contributing to lower resuspension;^240^ however, the effect diminishes as the pores become clogged, necessitating effective and regular rinsing.^241^ As with all nonexhaust sources, emissions from resuspension are lessened by reduced traffic volumes. Since regulations reducing speed and heavy vehicles in the fleet will reduce resuspension, the current trend toward heavier passenger vehicles is undesirable.

## 7 Discussion

Nonexhaust particle emissions arising from wear of brakes, tires, and the road surface, and the resuspension of road dust, are unregulated and exceed exhaust emissions in many jurisdictions. Despite this, owing to a lack of epidemiological and toxicological research, we do not have a clear picture of the health risk they pose. This calls for a multidisciplinary research effort to tailor effective and appropriate evidence-based legislation and abatement strategies to protect human health.

Quantitative data on the magnitude of NEE are sparse and highly uncertain owing to different methodological approaches and many complex variables including different vehicle fleets, environmental/meteorological determinants, driving styles (e.g., rural/urban/motorway), operational features (e.g., vehicle speed, acceleration/deceleration), and physical factors (e.g., vehicle mass, brake/tire/road material compositions, and properties). To gain a greater understanding of real-world concentrations to better inform exposure model development and validation for studies on human health, there is a need for a consensus on internationally consistent test setups, to ensure that measurement methods and results are repeatable and reproducible, enabling independent laboratories to assess NEE. To this end, the United Nations Economic Commission for Europe Working Party on Noise and Tyres (Groupe Rapporteur Bruit et Pneumatiques (GRBP)) and industry bodies are developing standardized test procedures to measure brake and tire particle mass and number emissions.^222,242^ At the time of writing, the Particle Measurement Program Working Group has recommended a minimum specification for measurement of brake particle emissions, but not a finalized protocol. Less progress has been made toward standardization of tire particle emissions. When it comes to measuring suspension of or sampling of road dust, methodologies (stationary using a vacuum^55,243^ or wet dust sampler;^244^ use of mobile laboratories sampling behind wheels^245,246^) are at an early stage of development with little or no harmonization.

Another major bottleneck preventing epidemiological studies from fully quantifying the health effects of NEE is the absence of accurate exposure assessment of often highly correlated source components within PM_2.5_ and PM_10_. Improving exposure estimates will require further investment into intensive sampling campaigns that capture detailed spatial and temporal profiles of NEE by measuring particle mass, size distribution, and chemical composition simultaneously at background locations and near road locations, close to where individuals live and commute.

Current methods to assess short-term exposure can identify nonexhaust sources as *mixtures of pollutants* generated by road dusts, brake wear, and tire wear. Although this indicator is useful for short-term epidemiological studies, precise estimates of *individual nonexhaust sources* are needed. These sources, which display a significant decay in concentrations from the roadside to urban background, may be better apportioned by use of data from personal exposure monitoring, near-roadway monitoring stations, or mobile monitoring that aims to characterize in-vehicle or on-road exposure. The development of effective biomarkers of NEE exposure in blood or urine would also significantly assist, but this is complex given that many of the candidate biomarkers are also associated with other urban stressors, other air pollution sources and poor diet.

For the assessment of long-term exposure, dispersion models have potential to provide PM concentrations attributable to nonexhaust sources. It is noteworthy and informative for future studies, that in most European study areas, elemental PM_10_ models perform better than PM_2.5_ ones for trace metals.^150^ More efforts are however needed to effectively disentangle the correlation with exposure to exhaust PM, as well as other pollutants and constituents thereof, and to generalize the model application more broadly beyond study areas. Future studies utilizing LUR should adopt larger sample sizes and longer monitoring periods to support the development models that can capture temporal variation. Hybrid LUR models that rely on precisely apportioned nonexhaust sources of PM mixtures and can incorporate source-specific predictor variables, such as nonexhaust dispersion model predictions, would be highly valuable for future epidemiological studies. Furthermore, the current changing pattern of vehicle emissions linked to improved abatement of engine exhaust pollutants may provide new opportunities for health effects studies.

To date, human experimental and panel studies have examined acute biochemical, inflammatory, and physiological responses to source specific components of ambient PM through examining responses in highly contrasting microenvironments and the underlying correlations between components and response.^247,248^ Findings from the limited number of epidemiological studies that focused specifically on certain NEE tracer elements or source factors suggest PM Zn and road dust source factors may be associated with acute and chronic cardiovascular outcomes, as well as birth outcomes. These studies have provided some insights, but the results are often and require further validation. There is also an absence of mechanistic work to place the associations observed into causal pathways relevant to disease etiology, progression, or exacerbation.

The toxicological evidence examining the relative hazard of exhaust derived PM versus that from other discrete nonexhaust sources is also limited but evolving. Several forms of NEE have the capacity to induce adverse effects in the lung.^192,201^ Now attention must turn to other organ systems and establish effects at real-world concentrations. There is a need for defined reference materials, reflecting NEE sources, but a consensus as to what these should be, given the heterogeneity of components within tire and brake wear, and road dust will need to be established first. Such a consensus should consider the need to focus on the toxicity of *all* size fractions of nonexhaust particles. Previous toxicological evidence has examined ambient PM samples collected from roadside, versus urban background locations, and therefore enriched with components of NEE. These studies have often made inferences about NEE components and a range of end points, usually related to the induction of acute inflammatory responses/injury or metabolic response profiles through correlation with PM components associated with NEE.^184,249^ Such approaches have similar limitations to those inherent in epidemiological studies, as many of the chemical components are highly correlated. While this literature, together with the mature toxicological literature on transition metals from the occupational and dietary based research,^250−253^ provides insight into the potential hazard of NEE, difficulties interpreting studies in relation to the relative toxicity of particles from nonexhaust sources should not be underestimated, as there is currently no consensus on the relative toxicity of the major components or sources of PM.^254,255^

Since many of the metals associated with nonexhaust PM can catalyze oxidation reactions, a consideration of their contribution to the oxidative potential (OP; the capacity of particulate pollution to cause damaging oxidative reactions) of ambient PM is warranted. Indeed, previous studies have indicated that they contribute significantly to this metric and a clear roadside increment in PM_2.5_ and PM_10_ OP has been demonstrated.^256^ A greater focus is also required on the nonmetal components of NEE, specifically the organic components derived from brake and tire wear, and trace elements within NEE should also not be excluded for consideration. Furthermore, given the capacity of cells and tissues to regulate metal uptake and store these ions in endogenous chelation proteins, such as ferritin and metallothionein, it is likely that their true hazard will not be reflected in acute exposure models and the accumulative impact of longer-term exposures will need to be addressed.

Currently it is unknown if there are of groups of individuals with higher susceptibility to the health effects of NEE and whether this would be similar to that of other air pollutants. Given the high content of Cu, Zn, and Fe in some NEE, individuals with genetic defects in the biological handing of these metals (e.g., individuals with hereditary hemochromatosis: the most common genetic disease in individuals of European ancestry^257^) would be assumed to be at greater risk. Fe, Zn, and Cu are also essential for microbial growth, and therefore future research should consider changes to the respiratory microbiome in individuals with depressed innate immunity.

Achieving the multidisciplinary elements of a research effort described above is an essential prerequisite to inform policy responses to NEE, such as legislating minimum low emission standard for brakes, tires, and/or road surface materials and/or resuspended road dust. Potential mitigation strategies include development of brake materials with reduced wear properties, identifying alternative materials to rubber for tires, on-vehicle brake/tire wear capture, the use of dust bindings/high vacuum sweepers combined with high-pressure washing to reduce resuspension, as well as overarching measures of lowering speed, promoting smoother driving behavior, and reducing vehicle mass. Further technical innovation behind such initiatives is encouraged. This must occur in tandem with studies that quantify the efficacy and safety of solutions, ensuring, for example, that new materials to reduce emissions are not associated with equivalent or heightened toxicity that could offset health benefits of this unregulated aspect of vehicular pollution that is forecast to continue to become ever more dominant in future years.
